# SERIOUS MENTAL ILLNESS IN MINNESOTA NURSING HOMES: THE ROLE OF RESIDENT AND FACILITY CHARACTERISTICS

**DOI:** 10.1093/geroni/igac059.2367

**Published:** 2022-12-20

**Authors:** Tetyana Shippee, Taylor Bucy, Weiwen Ng, Mark Woodhouse, Shekinah Fashaw-Walters, John Bowblis

**Affiliations:** University of Minnesota, Minneapolis, Minnesota, United States; University of Minnesota School of Public Health, Minneapolis, Minnesota, United States; University of Minnesota School of Public Health, Minneapolis, Minnesota, United States; University of Minnesota, Minneapolis, Minnesota, United States; University of Minnesota School of Public Health, Minneapolis, Minnesota, United States; Miami University, Oxford, Ohio, United States

## Abstract

Multiple studies have shown an increasing prevalence of adults with serious mental illness (SMI) in nursing homes. As adults with SMI age, the reality of care needs that span physical, medical, and psychosocial services necessitates further consideration of the role of comprehensive, ancillary mental health services in nursing homes (NH). Yet, little work examines characteristics of those with SMI, their care needs & the role of facility structural factors. Using the 2011-2017 Minimum Dataset (MDS) assessment data for Minnesota, we examined resident-level demographic characteristics of NH residents with and without SMI, and facility-level characteristics including quality of life (QoL), quality of care (QoC), and state recertification survey scores. We defined SMI as a diagnosis of bipolar disorder, schizophrenia or schizoaffective disorder, or psychotic conditions other than schizophrenia present on the reference assessment. Individuals admitted with SMI were younger, had better physical health, were more likely to be racial/ethnic minorities, and more likely to be admitted to a facility with a higher proportion of racial/ethnic minority residents. SMI-only admissions were concentrated in larger, for-profit facilities with a high-reliance on Medicaid. Lastly, SMI-only admissions were more likely to occur in facilities with lower QoL, QoC, and inspection scores. There is a growing need for behavioral health services in NHs, yet access to services is inadequate and lacks equity based on geography, race/ethnicity and other system-level disparities.

